# KAT2A Promotes the Progression of Renal Cell Carcinoma by Regulating the Succinylation of SERPINE2

**DOI:** 10.1002/kjm2.70211

**Published:** 2026-04-12

**Authors:** Chang‐Cheng Liu, Xue‐Dong Li

**Affiliations:** ^1^ The Second Affiliated Hospital of Harbin Medical University Harbin Heilongjiang China

**Keywords:** KAT2A, renal cell carcinoma, SERPINE2, Succinylation

## Abstract

Epithelial‐mesenchymal transition (EMT) and angiogenesis are critical drivers of renal cell carcinoma (RCC) progression, yet their upstream regulatory mechanisms remain incompletely understood. This study aimed to investigate the role of KAT2A in RCC and elucidate the underlying molecular mechanism by which it promotes tumor progression. To investigate KAT2A and SERPINE2 in RCC, we assessed their expression and downstream effects using real‐time quantitative PCR and western blot. EMT markers (E‐cadherin, N‐cadherin, Vimentin) and SERPINE2 succinylation were also measured. Functional assays (CCK‐8, colony formation, transwell, tube formation) evaluated cell viability, proliferation, migration, and angiogenesis. Co‐localization (immunofluorescence) and interaction (co‐immunoprecipitation) of KAT2A and SERPINE2 were confirmed. Finally, Ki‐67, KAT2A, and SERPINE2 expression in RCC tumors were examined via immunohistochemistry. We identified that KAT2A expression was significantly elevated in RCC tissues and correlated with poor patient prognosis. Functionally, KAT2A depletion markedly inhibited RCC cell proliferation, migration, EMT, and HUVECs angiogenesis in vitro, as well as suppressed tumor growth in vivo. Mechanistically, KAT2A directly interacted with SERPINE2 and promoted its succinylation at lysine 158 (K158). This post‐translational modification enhanced SERPINE2 protein stability, and SERPINE2 overexpression effectively reversed the tumor‐suppressive effects induced by KAT2A silencing. Our findings reveal that the KAT2A/SERPINE2 axis is a key regulator of RCC pathogenesis, identifying KAT2A‐mediated SERPINE2 succinylation as a novel mechanism and potential therapeutic target for RCC treatment.

**Abbreviations**
ATCC: American Type Culture Collection
CCK‐8: cell counting kit‐8
CHX: cycloheximide
Co‐IP: co‐immunoprecipitation
EMT: epithelial‐mesenchymal transition
FBS: fetal bovine serum
HUVECs: human umbilical vein endothelial cells
IHC: immunohistochemistry
KAT2A: lysine acetyltransferase 2A
RCC: renal cell carcinoma
RT‐qPCR: real‐time quantitative PCR
SD: standard deviation
SERPINE2: Serine protease inhibitor E2
Suc: succinylation
TCGA: The Cancer Genome Atlas

## 1 Introduction

Renal cell carcinoma (RCC) is the most common primary malignant tumor of the kidney, and it accounts for more than 85% of primary malignant kidney tumors [1]. The identified risk factors include tobacco use, obesity, hypertension, and long‐term exposure to environmental toxins. Radical surgery is the main treatment for early RCC. However, many patients with RCC are diagnosed at an advanced stage or with metastasis at the time of diagnosis, making treatment difficult [2]. Epithelial‐mesenchymal transition (EMT) refers to a complex process in which cells lose epithelial characteristics and acquire mesenchymal characteristics [3]. During EMT, cancer cells can invade distant tissues from the primary lesion through blood vessels and lymphatic vessels. EMT is often accompanied by the down‐regulation of epithelial marker E‐cadherin and the up‐regulation of interstitial marker N‐cadherin [4]. Meanwhile, angiogenesis is the main malignant phenotypic feature of tumors. New blood vessels not only provide nutrients for the proliferation of tumor cells but also provide an important route for tumor cells to further invade [5]. EMT and angiogenesis play central roles in the biological processes involved in RCC progression by enhancing tumor invasiveness and supporting neovascularization [6, 7, 8]. Therefore, understanding the molecular events that regulate EMT and vascular remodeling in RCC is critical for refining diagnostic markers and improving therapeutic strategies.

Serine protease inhibitor E2 (SERPINE2) is a member of the serpin family, which is known to regulate protease activity in the extracellular matrix. Its abnormal expression has been documented in a variety of cancers, and it is often associated with enhanced tumor cell mobility and metastasis [9]. Transcriptomics and single‐cell RNA sequencing analysis showed that SERPINE2 expression is increased in RCC tumor tissues compared to adjacent normal tissues [10]. Functional studies further showed that silencing SERPINE2 inhibited the proliferation and invasion of RCC cells, whereas forced overexpression promoted these malignant characteristics [11]. Additionally, SERPINE2 is also known to be involved in EMT activation by regulating matrix remodeling and downstream transcriptional programs [12, 13]. It has also been reported to affect tumor vascular remodeling by interacting with the angiogenesis pathway, suggesting that it plays a role in maintaining tumor‐associated neovascularization [14, 15]. These findings suggest that SERPINE2 is a potential contributor to RCC progression. However, whether it plays its specific tumor‐promoting role in RCC by regulating EMT and angiogenesis remains to be determined.

Succinylation (Suc) is a post‐translational modification that covalently adds succinyl groups to lysine residues to change the structure, charge state, and biochemical behavior of proteins. This modification is increasingly considered to be an important regulator of cell metabolism and tumor biology [16]. Dysregulation of succinylation has been observed in RCC and is thought to affect cancer‐related pathways, including mitochondrial function, signal transduction, and chromatin dynamics [17, 18]. Histone acetyltransferase lysine acetyltransferase 2A (KAT2A) has also been reported to have succinyltransferase activity, which may affect tumor progression through lysine succinylation [19]. There is evidence that it is involved in promoting cell proliferation, invasion, and metastasis in RCC models [20]. However, the specific molecular mechanism linking KAT2A to RCC progression remains unclear. Using the GPSuc (http://kurata14.bio.kyutech.ac.jp/GPSuc/index.php) database, we found several possible succinylation sites on SERPINE2, suggesting that KAT2A‐mediated succinylation may regulate the stability and function of SERPINE2. However, whether this regulatory mechanism contributes to the malignant progression of RCC has not been established, and further research is needed.

Based on the above evidence, we speculated that KAT2A promotes RCC progression by regulating the succinylation of SERPINE2. Thus, in this study, we investigated the functional consequences of KAT2A/SERPINE2 in RCC cells and examined the regulatory relationship between KAT2A and SERPINE2. The results of this study provide insights into the mechanism of RCC biology‐related succinylation pathways and emphasize the role of the previously uncharacterized KAT2A‐SERPINE2 axis in the progression of RCC, suggesting that the KAT2A/SERPINE2 axis may be a new predictive biomarker and therapeutic target for RCC.

## 2 Materials and Methods

### 2.1 Patient Samples

The 15 pairs of RCC tumor samples and normal adjacent tissues were obtained from The Second Affiliated Hospital of Harbin Medical University from July 2023 to September 2024. Tissues were frozen and maintained in liquid nitrogen for the following analysis. The project was approved by the Ethics Committee of The Second Affiliated Hospital of Harbin Medical University (Approval number: 2023‐Ethics‐022). Written informed consent was obtained from all patients.

### 2.2 Cell Culture and Transfection

The human proximal tubular epithelial cell line HK‐2 (American Type Culture Collection, ATCC, Manassas, VA, USA, CRL‐2190) and RCC cell lines OSRC‐2 (Chinese Academy of Sciences, Shanghai, China, GNHu41), 786‐O (ATCC, CRL‐1932), Caki‐1 (ATCC, HTB‐46), 769‐P (ATCC, CRL‐1933), and A498 (ATCC, HTB‐44) were used in this study. Cells were maintained in RPMI‐1640 (Gibco, Grand Island, NY, USA) or DMEM (Gibco) medium supplemented with 10% fetal bovine serum (FBS; Gibco) and 1% penicillin–streptomycin (Gibco) and cultured in a 5% CO_2_ humidified incubator at 37°C.

For transfection, shRNAs targeting KAT2A (sh‐KAT2A) and negative control (sh‐NC) were designed and cloned into the pLKO.1‐puro vector (Addgene, #8453). The full‐length SERPINE2 coding sequence was amplified and subcloned into the pcDNA3.1(+) vector (Invitrogen, Carlsbad, CA, USA, V79020) to generate the overexpression plasmid (pc‐SERPINE2 and pc‐KAT2A), while the pcDNA3.1(+) empty vector served as the negative control (pc‐NC). The wild‐type SERPINE2 expression plasmid (Flag‐SERPINE2‐WT) and the lysine‐to‐arginine mutant plasmid (Flag‐SERPINE2‐K158R) were purchased from GenePharma (Shanghai, China). Plasmids were transfected using Lipofectamine 3000 reagent (Invitrogen) for 48 h according to the manufacturer's protocol.

### 2.3 Cell Counting Kit‐8 (CCK‐8) Assay

Cells were plated in 96‐well plates at 3000 cells/well. At indicated time points (0, 24, 48, 72 h), 10 μL of CCK‐8 solution (Sigma, St. Louis, USA) was added per well and incubated for 2 h. Absorbance at 450 nm was measured using a microplate reader (Thermo Fisher Scientific, Waltham, MA, USA).

### 2.4 Colony Formation Assay

Transfected RCC cells were seeded in six‐well plates (500 cells/well). After 2 weeks of culture, cells were fixed with 10% methanol for 10 min, and then stained with 0.1% crystal violet (Beyotime, Shanghai, China) for 10 min. The colonies were then observed using a microscope (Olympus, Tokyo, Japan). The number of colonies was quantitatively analyzed using ImageJ software (National Institutes of Health, NIH, Bethesda, MD, USA).

### 2.5 Transwell Assay

Cell migration was assessed using Transwell chambers (8‐μm pore, Corning, NY, USA). Cells (2 × 10^4^ in 200 μL serum‐free medium) were seeded into the upper chambers, and 600 μL of medium with 10% FBS was added to the lower chambers. After 24 h incubation, cells on the lower surface of the upper chamber were fixed with methanol, stained with 0.1% crystal violet (Beyotime), and counted in five random fields under a microscope (Olympus).

### 2.6 Tube Formation Assay

First, 200 μL of Matrigel (Corning) was added to each well of a 24‐well plate, followed by polymerization at 37°C for 30 min. Subsequently, 2 × 10^4^ human umbilical vein endothelial cells (HUVECs, ATCC, PCS‐100‐013) in 200 μL of conditioned medium from sh‐KAT2A and/or pc‐SERPINE2 transfected RCC cells were introduced into each well and allowed to incubate at 37°C for 12 h. Capillary‐like structures were captured by phase‐contrast microscopy (Olympus). Branch point/Field was subsequently analyzed utilizing ImageJ software (NIH).

### 2.7 Total RNA Extraction and Real‐Time Quantitative PCR (RT‐qPCR)

Total RNA was isolated from tissues and cells using TRIzol reagent (Invitrogen). cDNA was synthesized using PrimeScript RT Master Mix (TaKaRa, Tokyo, Japan) following the manufacturer's instructions. RT‐qPCR was conducted using TB Green Premix Ex Taq II (TaKaRa) on an ABI7500 qPCR instrument (Thermo Fisher Scientific). Primers were synthesized with sequences as follows: KAT2A F: 5′‐CAGGGTGTGCTGAACTTTGTG‐3′, R: 5′‐TCCAGTAGTTAAGGCAGAGCAA‐3′; SERPINE2 F: 5′‐AAGAAACGCACTTTCGTGGC‐3′, R: 5′‐CCGTGGTAGGGCAGTTCAAT‐3′; GAPDH F: 5′‐AGGTCGGAGTCAACGGATTT‐3′, R: 5′‐TGACGGTGCCATGGAATTTG‐3′. GAPDH was used as an internal control. Relative transcription level was evaluated using the 2^−∆∆Ct^ method.

### 2.8 Western Blot

Cells and tissues were lysed in RIPA buffer (Beyotime) supplemented with protease inhibitor cocktail (Roche, Basel, Switzerland). Protein concentrations were determined using a BCA assay (Beyotime). Equal amounts of protein were resolved by SDS‐PAGE and transferred to PVDF membranes (Millipore, Billerica, MA, USA). After blocking in 5% skim milk for 1 h at room temperature, membranes were incubated overnight at 4°C with the following primary antibodies: KAT2A (Abcam, Cambridge, MA, USA, ab121147, 1:1000), SERPINE2 (Proteintech, Rosemont, IL, USA, 16515‐1‐AP, 1:1000), E‐cadherin (Cell Signaling Technology, CST, Danvers, MA, USA, 3195S, 1:1000), N‐cadherin (CST, 13116S, 1:1000), Vimentin (CST, 5741S, 1:1000), succinyllysine (PTM Biolabs, PTM‐401, 1:1000), and GAPDH (Abcam, ab9485, 1:1000). After washing, membranes were incubated with HRP‐conjugated secondary antibodies (#7074, 1:1000, CST) for 1 h. Signal detection was performed using ECL substrate (Beyotime). Protein bands were assessed using ImageJ software (NIH).

### 2.9 Co‐Immunoprecipitation (Co‐IP) Assay

Co‐IP was performed using Pierce Classic Magnetic IP/Co‐IP Kit (Thermo Fisher Scientific, 88,804). Briefly, cells were lysed in IP lysis buffer (Beyotime) with protease inhibitors, and lysates were incubated overnight with either anti‐KAT2A (Abcam, ab121147) or anti‐SERPINE2 (Proteintech, 16,515–1‐AP) at 4°C. The immune complexes were captured with Protein A/G magnetic beads (Thermo Fisher Scientific, 88,803), washed, and eluted for western blot detection.

### 2.10 Immunofluorescence Staining

Caki‐1 cells grown on glass coverslips were fixed with 4% paraformaldehyde for 20 min and permeabilized with 0.1% Triton X‐100 for 10 min. After blocking in 5% BSA (Sigma), cells were incubated overnight at 4°C with primary antibodies against KAT2A (Abcam, ab121147) and SERPINE2 (Proteintech, 16,515–1‐AP), followed by secondary antibodies conjugated to Alexa Fluor 488 or 594 (Invitrogen, A11034/A11032, 1:500) for 1 h. Nuclei were counterstained with DAPI, and coverslips were mounted with anti‐fade mounting medium. Images were acquired using a confocal microscope (Olympus).

### 2.11 Protein Stability Detection

Cells were treated with Cycloheximide (CHX, Sigma, C4859) at 10 μg/mL and harvested at 0, 2, 4, and 6 h. Proteins were extracted, and SERPINE2 levels were analyzed by western blot. Band intensity was quantified to assess protein half‐life.

### 2.12 Animal Xenografts Study

The 6‐week‐old BALB/c male nude mice were obtained from Hunan SJA Laboratory Animal Co. Ltd. (Changsha, China), which maintains the animals in pathogen‐free facilities. Nude mice were randomly divided into the sh‐NC group and sh‐KAT2A group (*n* = 6 mice per group). Briefly, Caki‐1 cells (6 × 10^6^) stably transfected with sh‐KAT2A or sh‐NC were suspended in 150 μL of PBS buffer and subcutaneously injected into the flanks of nude mice. The tumor growth of mice was monitored every 7 days for a total of 28 days. At the end of 4 weeks, all mice were sacrificed. The tumors were excised and weighed, and tumor tissues were collected for further analysis. All experimental protocols were conducted in accordance with the ARRIVE 2.0 guidelines and approved by the Institutional Animal Care and Use Committee (IACUC) of The Second Affiliated Hospital of Harbin Medical University (Approval Number: 2023‐Ethics‐022).

### 2.13 Immunohistochemistry (IHC) Analysis

Tumor paraffin sections (4 μm thick) were dewaxed, rehydrated, and incubated with H_2_O_2_. After blocking in a blocking solution containing 5% BSA, sections were incubated with primary antibodies against Ki‐67 (Abcam, ab15580), KAT2A (Abcam, ab121147), SERPINE2 (Proteintech, 16,515–1‐AP), VEGFA (Abcam, ab46154), and CD34 (Abcam, ab81289) at 4°C overnight. After washing, sections were incubated with secondary antibody for 1 h at room temperature and then developed using DAB, counterstained with hematoxylin, dehydrated, and mounted. Images were observed under a light microscope (Olympus). Microvessel density was evaluated according to a previously established method [21]. Specifically, the number of CD34‐positive endothelial cells or cell clusters was counted under light microscopy and recorded as the microvessel density value.

### 2.14 Statistical Analysis

The Shapiro–Wilk normality test was performed to determine whether the data were normally distributed. Data were expressed as mean ± standard deviation (SD) and analyzed using SPSS 22.0 (IBM, Armonk, NY, USA) and GraphPad Prism 8.0 (La Jolla, CA, USA). All cell experiments were performed in triplicate (biological replicates). Two‐group comparisons were assessed using the two‐tailed unpaired Student's *t*‐test, while comparisons among multiple groups were evaluated using the one‐way ANOVA followed by Tukey's post hoc test. Pearson correlation analysis was used to analyze the relationship between KAT2A and SERPINE2 protein in RCC tissues. A *p* value less than 0.05 was considered statistically significant.

## 3 Results

### 3.1 KAT2A Was Increased in RCC and Correlated With Poor Prognosis

**FIGURE 1:**
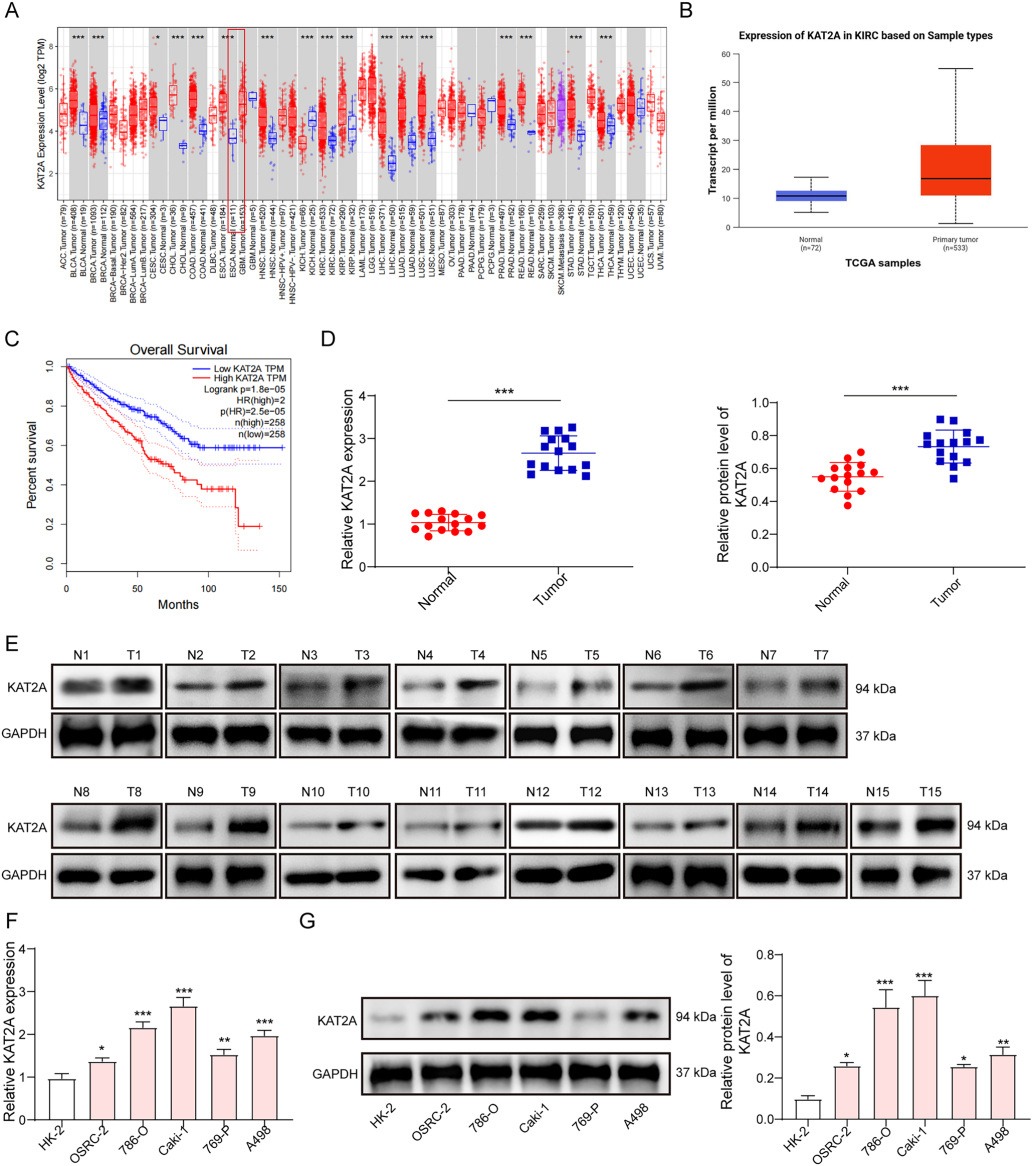
KAT2A was increased in RCC and correlated with poor prognosis. (A) TIMER2.0 analysis of KAT2A expression across multiple cancer types. (B) UALCAN analysis of KAT2A mRNA levels in RCC tissues versus normal kidney tissues (TCGA‐KIRC cohort). (C) Kaplan–Meier plot showing overall survival of RCC patients with high versus low KAT2A expression. (D, E) RT‐qPCR and western blot analysis of KAT2A mRNA and protein levels in paired RCC and adjacent normal tissues (*n* = 15). (F, G) RT‐qPCR and western blot analysis of KAT2A mRNA and protein levels in RCC cell lines and HK‐2 cells. Data were shown as mean ± SD from three independent experiments. *p < 0.05, **p < 0.01, ***p < 0.001. KAT2A: Lysine acetyltransferase 2A. RCC: Renal cell carcinoma. RT‐qPCR: Reverse transcription‐quantitative polymerase chain reaction.

To evaluate the expression profile of KAT2A in human cancers, we first analyzed pan‐cancer RNA‐seq data using the TIMER2.0 platform (http://timer.cistrome.org/). KAT2A was found to be upregulated in a variety of tumors, especially in RCC (Figure 1A). This observation was supported by the UALCAN database (https://ualcan.path.uab.edu/analysis.html), which showed that KAT2A expression in RCC tissues was significantly higher than that in adjacent tissues (Figure 1B). Survival analysis based on The Cancer Genome Atlas (TCGA) dataset further showed that high expression of KAT2A was associated with shorter overall survival, indicating that KAT2A had a negative impact on prognosis (Figure 1C). Next, we performed RT‐qPCR and western blot analysis on 15 paired RCC and adjacent normal tissues. The mRNA and protein levels of KAT2A were markedly elevated in tumor specimens (Figure 1D,E). The analysis of TCGA database revealed no significant correlation between KAT2A expression levels and the key clinical characteristics of RCC. Specifically, KAT2A expression did not increase with higher tumor stage, advanced TNM classification, or the presence of lymph node metastasis (Figure S1A–C). Consistent with these findings, the analysis of our in‐house cohort of clinical RCC specimens yielded similar results, showing no significant association between KAT2A expression and clinicopathological features or metastatic potential. Consequently, these data from our clinical samples are not presented in the main figures. Subsequently, we examined KAT2A expression across multiple RCC cell lines. Compared to HK‐2 cells, mRNA and protein levels of KAT2A were increased in RCC cell lines, especially in 786‐O and Caki‐1 cells (Figure 1F,G). Therefore, these two cell lines were selected for subsequent experiments. These results demonstrate that high expression of KAT2A in RCC is related to poor prognosis.

### 3.2 KAT2A Knockdown Inhibited EMT of RCC Cells and Angiogenesis of HUVECs

**FIGURE 2:**
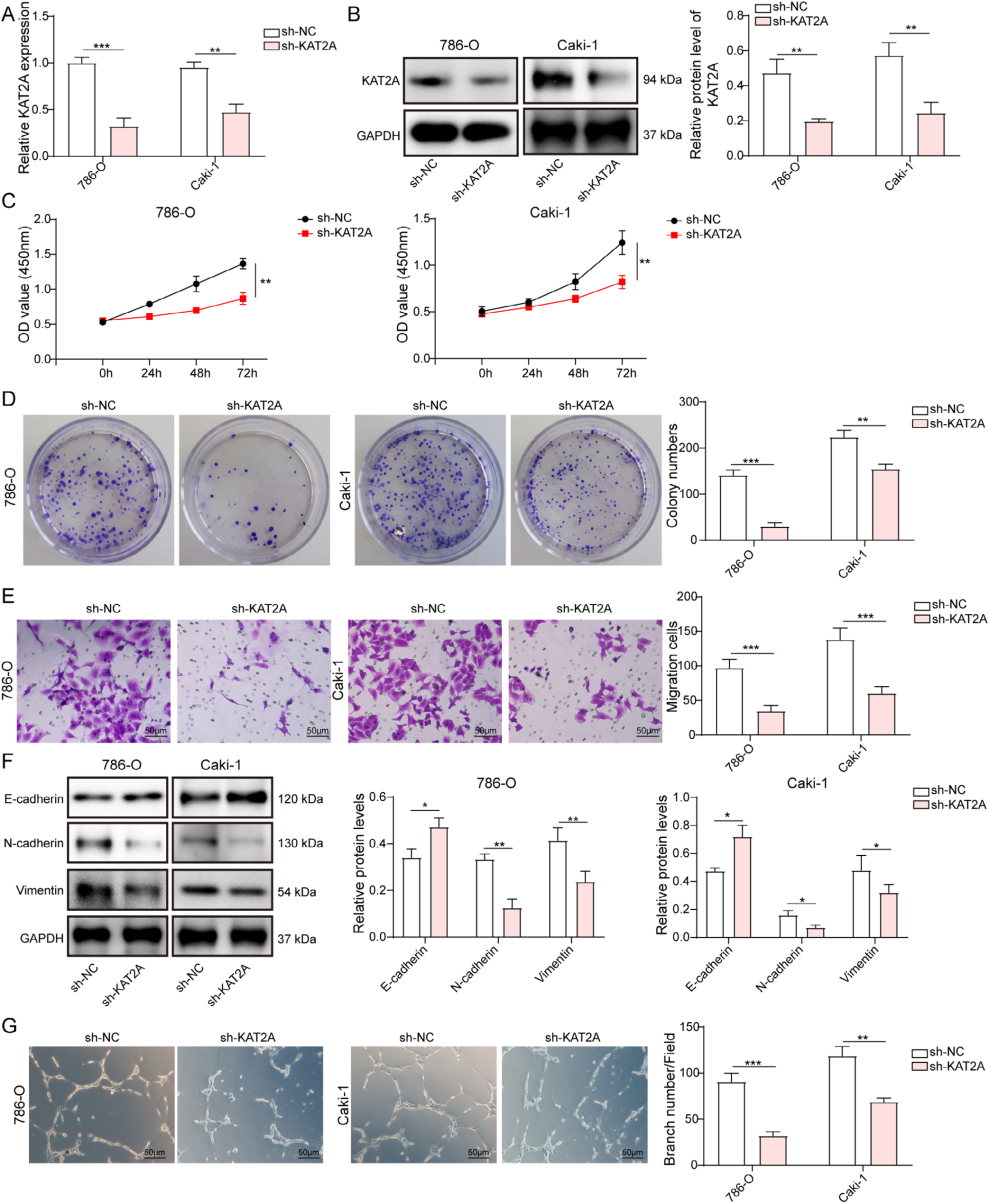
KAT2A knockdown inhibited EMT of RCC cells and angiogenesis of HUVECs. 786‐O and Caki‐1 cells were transfected with sh‐NC or sh‐KAT2A. (A, B) Confirmation of KAT2A knockdown efficiency by RT‐qPCR and western blot. (C) CCK‐8 analysis of cell proliferation. (D) Colony formation assay evaluated cell proliferation. (E) Transwell detection of cell migration. (F) Expression of EMT‐related proteins (E‐cadherin, N‐cadherin, and Vimentin) was determined by western blot. (G) Tube formation assay tested angiogenesis using HUVECs cultured in conditioned medium from sh‐KAT2A or sh‐NC cells. Data were shown as mean ± SD from three independent experiments. *p < 0.05, **p < 0.01, ***p < 0.001. KAT2A: Lysine acetyltransferase 2A. RT‐qPCR: Reverse transcription‐quantitative polymerase chain reaction. CCK‐8: Cell counting kit‐8. EMT: Epithelial‐mesenchymal transition. HUVECs: Human umbilical vein endothelial cells.

Next, we investigated the effects of KAT2A on EMT of RCC cells and angiogenesis of HUVECs. KAT2A knockdown was achieved in 786‐O and Caki‐1 cells using shRNA. After knocking down KAT2A, mRNA and protein levels of KAT2A were decreased significantly (Figure 2A,B). The CCK‐8 assay demonstrated that cell proliferation was inhibited after knockdown of KAT2A (Figure 2C). After silencing KAT2A, the number of cloned cells decreased significantly (Figure 2D). Meanwhile, Transwell assay indicated that KAT2A depletion greatly decreased the amount of migrated cells (Figure 2E). Additionally, KAT2A downregulation obviously suppressed the protein expression of N‐cadherin and Vimentin, but increased the expression of E‐cadherin (Figure 2F). Furthermore, we evaluated the angiogenesis using HUVECs cultured in conditioned medium from sh‐KAT2A or sh‐NC cells. Compared to the sh‐NC group, KAT2A silencing significantly inhibited the angiogenesis of HUVECs (Figure 2G). Consistently, overexpression of KAT2A in OSRC‐2 cells (Figure S2A,B) promoted cell proliferation (Figure S2C), colony formation (Figure S2D), migration (Figure S2E), and induced EMT, as evidenced by the increased N‐cadherin and Vimentin expression and decreased E‐cadherin expression (Figure S2F). Moreover, the conditioned medium from the KAT2A‐overexpressing OSRC‐2 cells enhanced the angiogenesis of HUVECs (Figure S2G). These results therefore imply that KAT2A promotes RCC cell EMT and HUVECs angiogenesis.

### 3.3 KAT2A Stabilized SERPINE2 Through Lysine Succinylation

**FIGURE 3:**
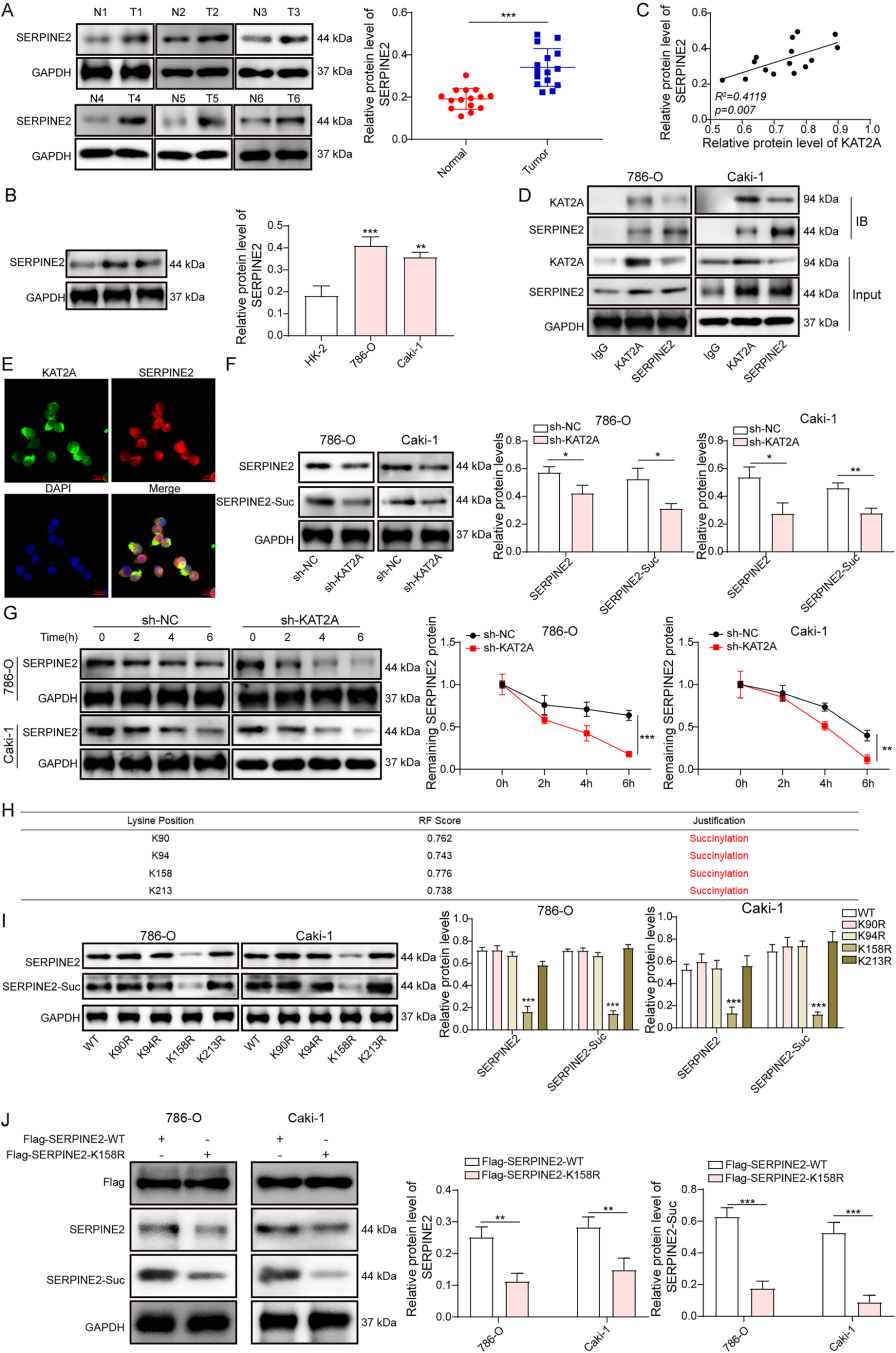
KAT2A stabilizes SERPINE2 through succinylation. (A) Western blot analysis of SERPINE2 protein levels in paired RCC and adjacent normal tissues (*n* = 15). (B) Western blot analysis of SERPINE2 protein levels in RCC cell lines and HK‐2 cells. (C) Correlation between SERPINE2 and KAT2A protein levels across 15 RCC samples. (D) Co‐IP assay verified that KAT2A protein interacted with SERPINE2 protein in RCC cells. (E) Immunofluorescence staining confirmed co‐localization of KAT2A and SERPINE2 in Caki‐1 cells. (F) Succinylation levels of SERPINE2 following KAT2A knockdown were detected by western blot. (G) CHX experiment was used to detect the stability of SERPINE2 protein. (H) GPSuc predicted succinylation sites on SERPINE2. (I) RCC cells were transfected with SERPINE2 mutants. SERPINE2 and SERPINE2‐Suc protein levels were determined by western blot. (J) Western blot analysis of Flag‐tagged SERPINE2 protein expression and its succinylation status in RCC cells transfected with Flag‐SERPINE2‐WT or Flag‐SERPINE2‐K158R mutant plasmids. Data were shown as mean ± SD from three independent experiments. *p < 0.05, **p < 0.01, ***p < 0.001. KAT2A: Lysine acetyltransferase 2A. SERPINE2: Serine protease inhibitor E2. Co‐IP: Co‐immunoprecipitation. CHX: Cycloheximide. RCC: Renal cell carcinoma.

Considering that SERPINE2 is upregulated in RCC and is associated with tumor progression [10], we questioned whether it may be functionally associated with KAT2A. We discovered that SERPINE2 protein expression was significantly increased in RCC tissues compared with adjacent tissues (Figure 3A). Similarly, compared with HK‐2 cells, the SERPINE2 protein level in RCC cells was greatly increased (Figure 3B). Notably, KAT2A protein was positively correlated with SERPINE2 protein in RCC tissues (Figure 3C). Furthermore, the Co‐IP assay confirmed that KAT2A interacted with SERPINE2 in RCC cells (Figure 3D). Meanwhile, immunofluorescence staining showed co‐localization of KAT2A and SERPINE2 in Caki‐1 cells (Figure 3E). Interestingly, when KAT2A was silenced, the SERPINE2 protein level decreased and its succinylated form also decreased sharply (Figure 3F). Subsequently, we monitored SERPINE2 degradation after CHX treatment and found that knockdown of KAT2A reduced the stability of SERPINE2 protein in RCC cells (Figure 3G). Using the GPSuc database (http://kurata14.bio.kyutech.ac.jp/GPSuc/index.php), we identified four predicted succinylation sites on SERPINE2 (K90, K94, K158, and K213), all with a high succinylation potential (Figure 3H). RCC cells were co‐transfected with pc‐KAT2A and SERPINE2 mutants. Compared with SERPINE2‐WT plasmid, the expressions of SERPINE2 and SERPINE2‐Suc protein levels were significantly decreased after SERPINE2‐K158R mutant plasmid transfection, indicating that SERPINE2 was succinylated at K158 (Figure 3I). In addition, compared with the protein levels in cells transfected with the Flag‐SERPINE2‐WT plasmid, the protein levels of both SERPINE2 and SERPINE2‐Suc were significantly reduced in cells transfected with the Flag‐SERPINE2‐K158R mutant plasmid. This result confirmed that SERPINE2 was succinylated specifically at the K158 residue (Figure 3J). Altogether, the above data indicate that KAT2A stabilizes SERPINE2 through the succinylation of SERPINE2.

### 3.4 SERPINE2 Mediated the Oncogenic Effects of KAT2A in RCC Cells

**FIGURE 4:**
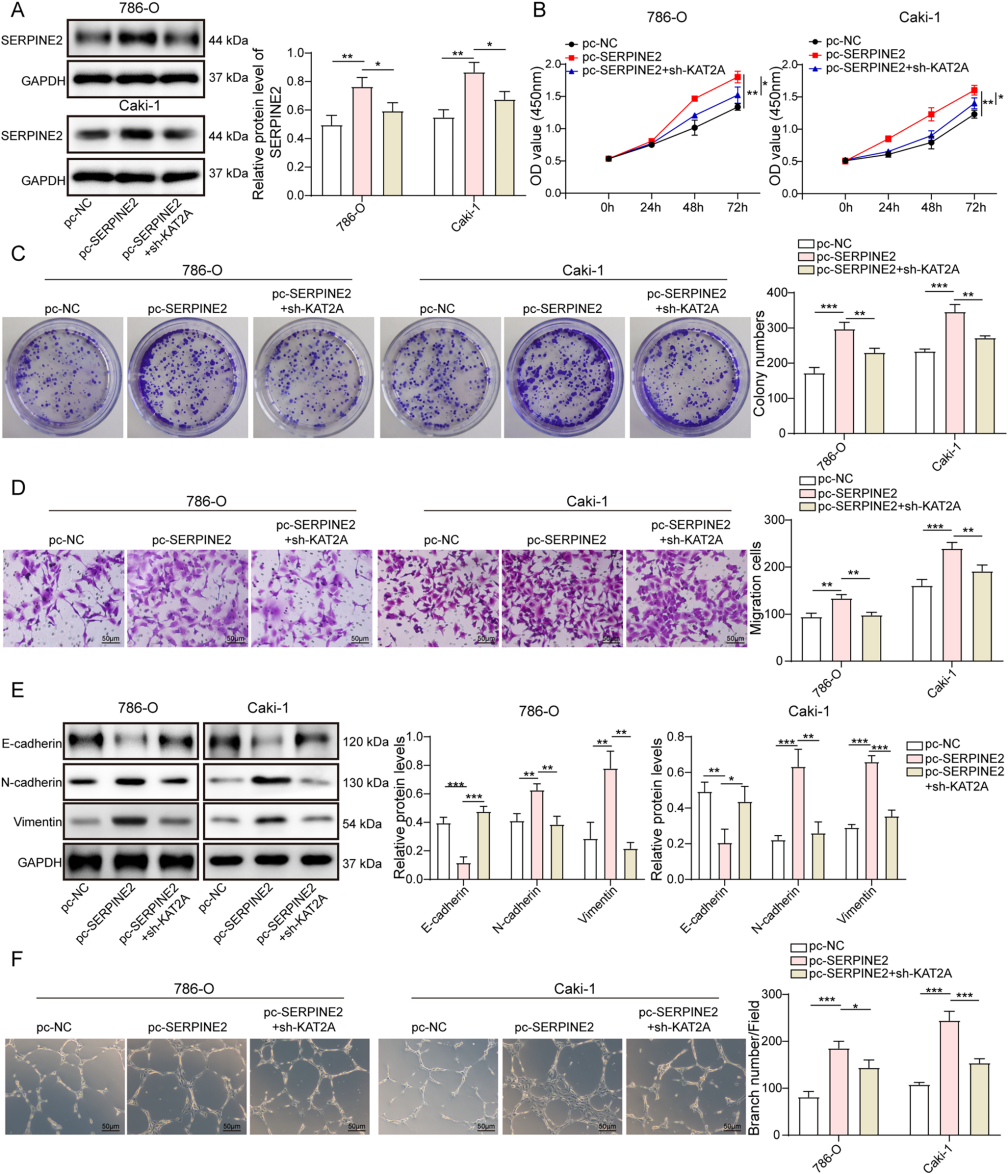
SERPINE2 mediated the oncogenic effects of KAT2A in RCC cells. SERPINE2 was overexpressed in 786‐O and Caki‐1 cells, with or without KAT2A knockdown. (A) Western blot analysis of SERPINE2 levels. (B) CCK‐8 analysis of cell proliferation. (C) Colony formation assay evaluated cell proliferation. (D) Transwell detection of cell migration. (E) EMT‐related proteins (E‐cadherin, N‐cadherin, and Vimentin) were determined by western blot. (F) Tube formation assay tested angiogenesis using HUVECs cultured in conditioned medium from RCC cells. Data were shown as mean ± SD from three independent experiments. *p < 0.05, **p < 0.01, ***p < 0.001. KAT2A: Lysine acetyltransferase 2A. SERPINE2: Serine protease inhibitor E2. CCK‐8: Cell counting kit‐8. EMT: Epithelial‐mesenchymal transition. HUVECs: Human umbilical vein endothelial cells. RCC: Renal cell carcinoma.

Then, we studied whether SERPINE2 plays a role in the carcinogenic effect of KAT2A in RCC. To this end, we overexpressed SERPINE2 in 786‐O and Caki‐1 cells with or without KAT2A knockdown. Western blot confirmed the successful upregulation of SERPINE2 in RCC cells with pc‐SERPINE2, and this increase was partially reversed by KAT2A silencing (Figure 4A). Functionally, the CCK‐8 assay indicated that the overexpression of SERPINE2 significantly enhanced cell proliferation, which was abolished by the suppression of KAT2A (Figure 4B). Compared with the pc‐NC group, overexpressed SERPINE2 greatly increased the number of cell clones, whereas the further knockdown of KAT2A reduced the number of cell clones (Figure 4C). Additionally, SERPINE2 upregulation remarkably promoted cell migration, while co‐transfection of sh‐KAT2A weakened this effect (Figure 4D). Furthermore, the overexpression of SERPINE2 dramatically increased N‐cadherin and Vimentin expression but decreased E‐cadherin expression; however, KAT2A silencing further abolished the dysregulation of these proteins (Figure 4E). Afterwards, we evaluated angiogenesis using HUVECs cultured in conditioned medium from RCC cells of different groups. Compared with the pc‐NC group, tube formation in the pc‐SERPINE2 group was significantly increased after co‐culture with HUVECs, which was overturned by sh‐KAT2A (Figure 4F). Together, these data indicate that KAT2A promotes the EMT of RCC cells and the angiogenesis of HUVECs by upregulating SERPINE2.

### 3.5 KAT2A Depletion Inhibited In Vivo Tumor Growth by Downregulating SERPINE2

**FIGURE 5:**
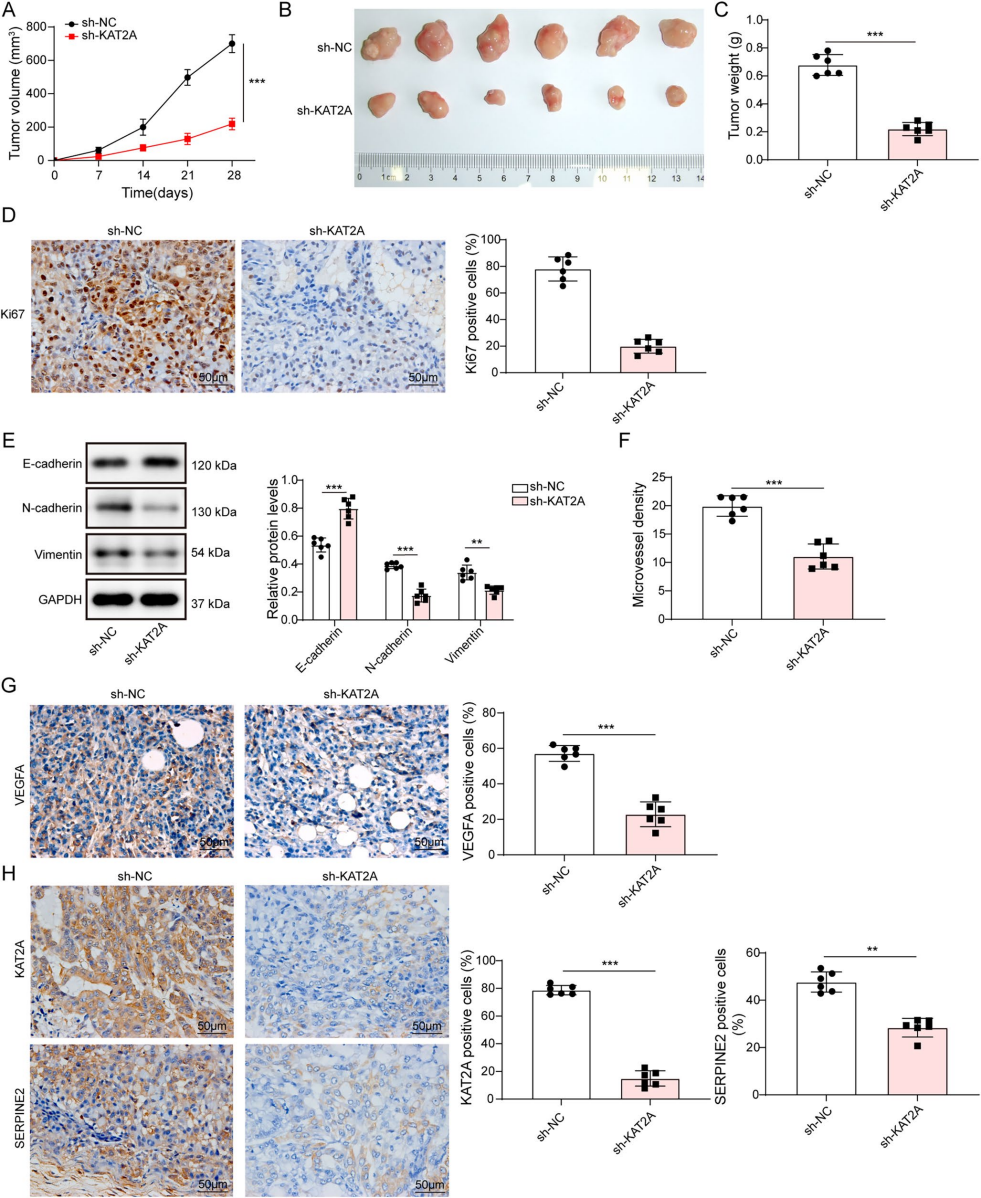
KAT2A depletion inhibited in vivo tumor growth by downregulating SERPINE2.(A‐C) Tumor volume curve, tumor weight, and representative images of xenografts from nude mice injected with sh‐NC or sh‐KAT2A Caki‐1 cells. (D) IHC was used to detect Ki‐67 levels in tumor tissues. (E) EMT‐related proteins in tumor tissues were determined by western blot. (F) Detection of microvessel density. (G) VEGFA expression in tumor tissues was evaluated by IHC staining. (H) IHC staining of KAT2A and SERPINE2 in tumor tissues. Values were expressed as mean ± SD. *n* = 6, **p < 0.01, ***p < 0.001. KAT2A: Lysine acetyltransferase 2A. SERPINE2: Serine protease inhibitor E2. IHC: Immunohistochemistry. EMT: Epithelial‐mesenchymal transition. VEGFA: Vascular endothelial growth factor A.

To evaluate the in vivo correlation of the KAT2A‐SERPINE2 axis, we established a xenograft model using Caki‐1 cells transfected with sh‐NC or sh‐KAT2A. After subcutaneous implantation in nude mice, tumor growth was observed after 28 days. Mice injected with sh‐KAT2A cells formed smaller tumors than those injected with sh‐NC cells (Figure 5A). At the end of the experiment, both tumor size and weight were significantly lower in the KAT2A knockdown group (Figure 5B,C). Additionally, IHC staining revealed that KAT2A downregulation reduced Ki‐67 expression in tumor tissues (Figure 5D). Consistent with the results of in vitro studies, we also found that silencing KAT2A led to an increase in E‐cadherin and a decrease in N‐cadherin and Vimentin in tumor tissues, indicating a reversal of the mesenchymal phenotype (Figure 5E). In addition, knockdown of KAT2A significantly reduced microvessel density (Figure 5F) and VEGFA expression (Figure 5G) in tumor tissues, suggesting inhibition of angiogenesis. Importantly, tissue sections stained with KAT2A and SERPINE2 showed parallel downregulation of these two proteins in tumors with KAT2A depletion (Figure 5H). Taken together, these findings reveal that KAT2A promotes RCC tumor growth by upregulating SERPINE2 in mice.

## 4 Discussion

Local tumor progression and distant metastasis of RCC are the main causes of death in patients with RCC. EMT and angiogenesis are two basic processes that drive the malignant progression of RCC, which help to enhance invasion, metastasis, and tumor‐associated angiogenesis [22, 23]. Although multiple signaling pathways are involved in their regulation, post‐translational modifications such as succinylation remain poorly understood in this context [24]. Previous studies have shown that EMT is not only a phenotypic change but also closely related to metabolic remodeling and extracellular matrix dynamics [25, 26], and that tumor‐derived factors such as SERPINE2 can orchestrate both EMT and endothelial activation [12]. Building upon this framework, our study identifies a novel regulatory axis wherein KAT2A, acting as a succinyltransferase, drives EMT and angiogenesis in RCC by stabilizing SERPINE2 through lysine succinylation.

SERPINE2 is a member of the serine protease inhibitor superfamily, and it plays an important role in hemostasis, thrombosis, and vascular biology [27]. More and more evidence suggests that SERPINE2 is a key driver of RCC invasiveness. A recent single‐cell RNA sequencing study showed that SERPINE2 is highly expressed in advanced RCC tissues and is closely related to metastatic potential and poor prognosis [10]. Functional data in this study showed that silencing SERPINE2 significantly reduced the proliferation and invasion of RCC cells, while its overexpression promoted tumor progression phenotype [10]. These findings are consistent with our observations that SERPINE2 protein expression was significantly increased in RCC tissues and cells. To support this, a separate analysis of TCGA data also identified SERPINE2 as a hub gene associated with poor RCC prognosis [28]. In addition, recent proteomics studies have shown that SERPINE2 may contribute to extracellular matrix remodeling and angiogenesis, both of which are hallmarks of aggressive RCC [29]. We discovered that KAT2A protein is positively correlated with SERPINE2 in RCC tissues and that it directly interacts with it. Crucially, we demonstrated for the first time that KAT2A enhances SERPINE2 protein stability by catalyzing its succinylation at the specific lysine 158 (K158) residue. This modification is functionally significant, as it underpins the ability of SERPINE2 to promote EMT markers, cell proliferation, migration, and angiogenesis in vitro and in vivo. As such, our work extends beyond merely confirming SERPINE2's tumor‐promoting role; it establishes KAT2A‐mediated lysine succinylation as a critical upstream regulatory mechanism controlling SERPINE2 stability and oncogenic function in RCC.

Succinylation is a new post‐translational modification of protein discovered in recent years. It is mainly a process in which succinyl coenzyme A transfers succinylated groups to lysine through enzymatic or non‐enzymatic methods [30]. Succinylation modification has been shown to be elevated in many tumors [31]. Although several global succinylated proteome studies have identified metabolic proteins that undergo succinylation in RCC tissues [32, 33], the regulatory effect of this modification on EMT and angiogenesis‐related effectors in RCC has not previously been addressed. KAT2A has historically been defined as a histone acetyltransferase and has recently been found to be involved in catalyzing lysine succinylation on non‐histone substrates [34]. KAT2A plays an important role in the occurrence and development of various cancers, where it supports tumor growth and invasiveness through different nuclear and cytoplasmic mechanisms [35, 36]. A recent study showed that KAT2A is an oncogenic chromatin modifier that promotes RCC progression by inducing MCT1 expression [20]. We provide the first evidence that KAT2A enhances succinylation and stabilizes the extracellular matrix regulator SERPINE2, thereby linking this modification directly with pro‐tumorigenic phenotypes. This finding positions the KAT2A‐SERPINE2 succinylation axis as a potential mechanistic pathway and a promising therapeutic target for RCC, warranting further exploration for diagnostic and intervention strategies.

Our findings also open new questions for future research. Although we focused on succinylation, KAT2A is a known acetyltransferase, and other acylations like lactylation are gaining prominence in cancer biology [37, 38]. An important and open question is whether there is crosstalk between different lysine acylations (e.g., acetylation, lactylation) in regulating SERPINE2 function or KAT2A activity in RCC. It is plausible that KAT2A‐mediated acetylation or competitive dynamics between modifications could fine‐tune SERPINE2 stability or interaction networks. Investigating this potential acylation crosstalk could provide a more comprehensive understanding of the nuanced regulatory landscape governing RCC progression.

**FIGURE 6:**
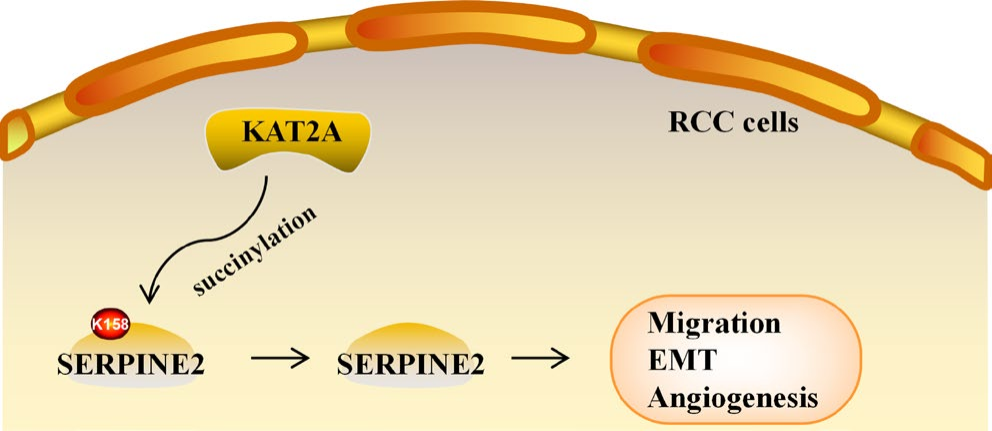
Summary diagram of this study. KAT2A promotes the progression of RCC by regulating the succinylation of SERPINE2. KAT2A: Lysine acetyltransferase 2A. SERPINE2: Serine protease inhibitor E2. RCC: Renal cell carcinoma.

In summary, we demonstrated that KAT2A stabilizes SERPINE2 through succinylation of lysine 158 and promotes EMT and angiogenesis, thereby driving the progress of RCC (Figure 6). Our results suggest that the KAT2A‐SERPINE2 axis represents a potential mechanistic pathway and prospective therapeutic target warranting further validation. However, this study has several limitations that should be acknowledged. First, the orthotopic transplantation/metastasis models and Kaplan–Meier survival curves lack validation. Second, although we identified lysine 158 as a key succinylation site on SERPINE2 and demonstrated its importance for protein stability, the specific succinyltransferase activity of KAT2A was not biochemically recombined in vitro. It is not clear whether KAT2A directly modifies SERPINE2 or acts via an intermediary complex. Additionally, although we confirmed the stability and downstream phenotype of SERPINE2 in RCC cell lines and xenograft tumors, we did not evaluate whether KAT2A‐SERPINE2 regulation occurs in primary patient‐derived cell or organoid models, which may better preserve tumor heterogeneity. Finally, this study focused on the effect of succinylation dependence, but whether KAT2A‐mediated acetylation or crosstalk between lysine modifications also contributes to the function of SERPINE2 in RCC remains to be explored.

## Ethics Statement

All procedures have been approved by the Institutional Animal Care and Use Committee (IACUC) of The Second Affiliated Hospital of Harbin Medical University (2023‐Ethics‐022). The author confirms all animal experiments were conducted in accordance with the ARRIVE 2.0 guidelines and the U.K. Animals (Scientific Procedures) Act, 1986 and associated guidelines, the EU Directive 2010/63/EU for animal experiments, or the National Institutes of Health Guide for the Care and Use of Laboratory Animals (NIH Publications No. 8023, revised 1978).

## Conflicts of Interest

The authors declare no conflicts of interest.

## Supporting information


**Figure S1:** Association of KAT2A expression with clinicopathological features in RCC based on TCGA data. KAT2A: Lysine acetyltransferase 2A. RCC: Renal cell carcinoma.
**Figure S2:** KAT2A overexpression promoted EMT of RCC cells and angiogenesis of HUVECs. OSRC‐2 cells were transfected with pc‐NC or pc‐KAT2A. (A, B) Confirmation of KAT2A overexpression efficiency by RT‐qPCR and western blot. (C) CCK‐8 analysis of cell proliferation. (D) Colony formation assay evaluated cell proliferation. (E) Transwell detection of cell migration. (F) Expression of EMT‐related proteins was determined by western blot. (G) Tube formation assay tested angiogenesis using HUVECs cultured in conditioned medium from control or KAT2A‐overexpressing OSRC‐2 cells. Data were shown as mean ± SD from three independent experiments. *p < 0.05, **p < 0.01, ***p < 0.001. KAT2A: Lysine acetyltransferase 2A. RT‐qPCR: Reverse transcription‐quantitative polymerase chain reaction. CCK‐8: Cell counting kit‐8. EMT: Epithelial‐mesenchymal transition. HUVECs: Human umbilical vein endothelial cells.

## Data Availability

The data that support the findings of this study are available from the corresponding author upon reasonable request.
