# Supplementary material for: KAT2A Promotes the Progression of Renal Cell Carcinoma by Regulating the Succinylation of SERPINE2
Source: Kaohsiung J Med Sci. 2026 Apr 12:e70211. Online ahead of print. doi: 10.1002/kjm2.70211 (PMC13399823; doi:10.1002/kjm2.70211)
Supplement: Supplementary file 1 — Figure S1: Association of KAT2A expression with clinicopathological features in RCC based on TCGA data. KAT2A: Lysine acetyltransferase 2A. RCC: Renal cell carcinoma. Figure S2: KAT2A overexpression promoted EMT of RCC cells and angiogenesis of HUVECs. OSRC‐2 cells were transfected with pc‐NC or pc‐KAT2A. (A, B) Confirmation of KAT2A overexpression efficiency by RT‐qPCR and western blot. (C) CCK‐8 analysis of cell proliferation. (D) Colony formation assay evaluated cell proliferation. (E) Transwell detection of cell migration. (F) Expression of EMT‐related proteins was determined by western blot. (G) Tube formation assay tested angiogenesis using HUVECs cultured in conditioned medium from control or KAT2A‐overexpressing OSRC‐2 cells. Data were shown as mean ± SD from three independent experiments. *p < 0.05, **p < 0.01, ***p < 0.001. KAT2A: Lysine acetyltransferase 2A. RT‐qPCR: Reverse transcription‐quantitative polymerase chain reaction. CCK‐8: Cell counting kit‐8. EMT: Epithelial‐mesenchymal transition. HUVECs: Human umbilical vein endothelial cells. [file KJM2-9999-e70211-s001.doc]

**Supplementary Materials.**
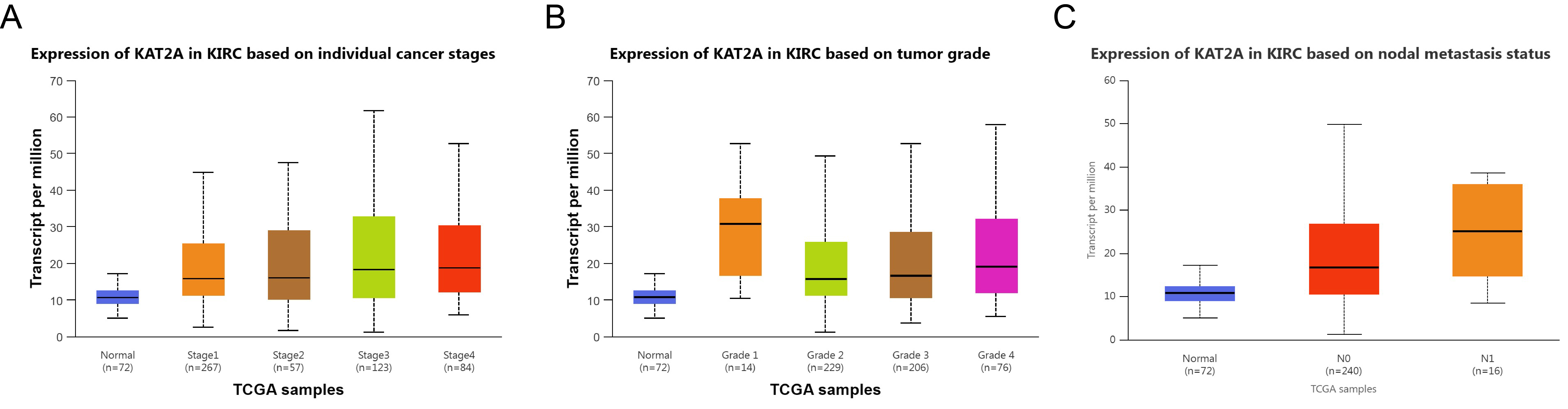


**Supplementary Figure 1. Association of KAT2A expression with clinicopathological features in RCC based on TCGA data.** KAT2A: Lysine acetyltransferase 2A. RCC: Renal cell carcinoma.


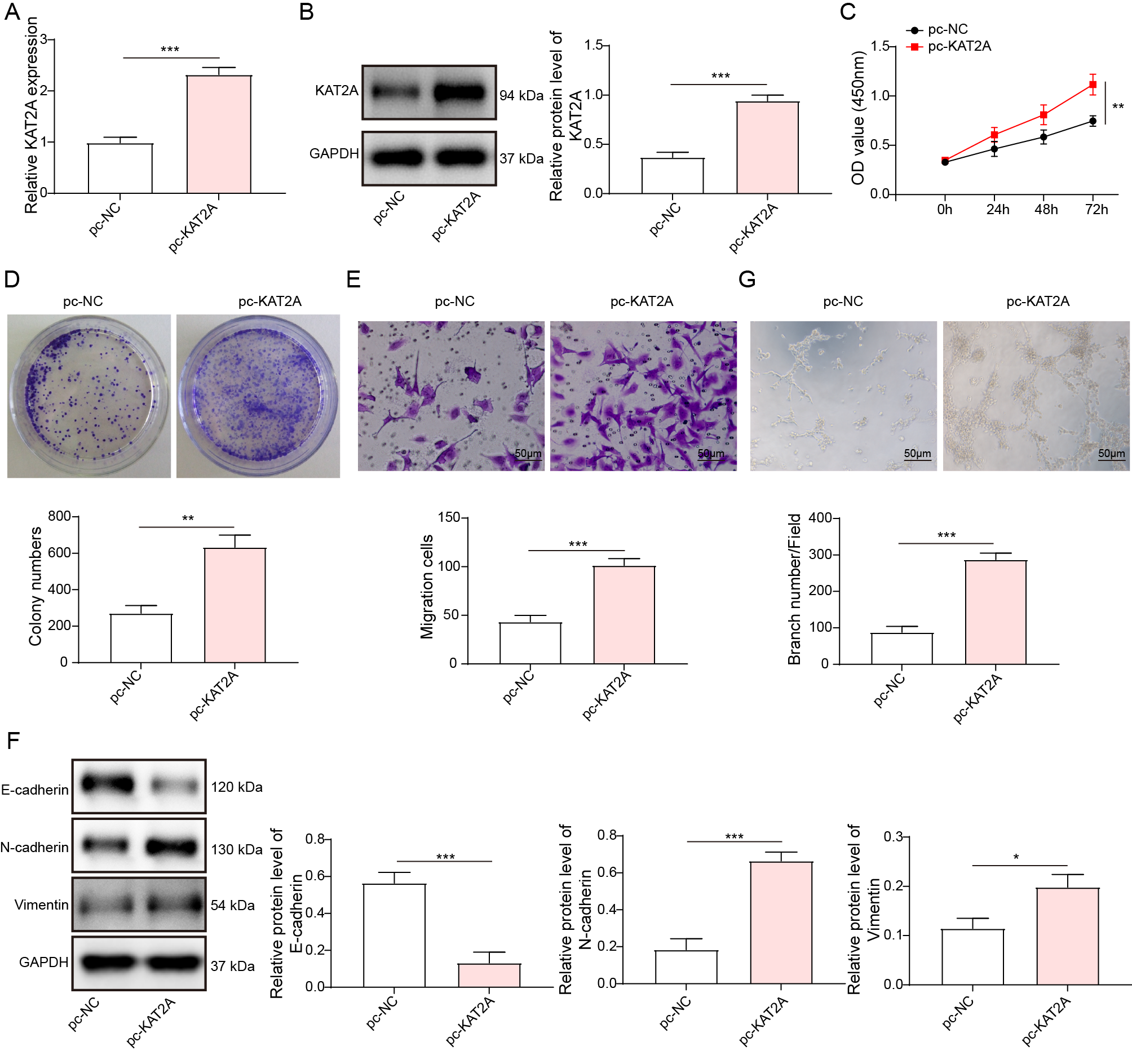


**Supplementary Figure 2. KAT2A overexpression promoted EMT of RCC cells and angiogenesis of HUVECs.** OSRC-2 cells were transfected with pc-NC or pc-KAT2A. (A, B) Confirmation of KAT2A overexpression efficiency by RT-qPCR and western blot. (C) CCK-8 analysis of cell proliferation. (D) Colony formation assay evaluated cell proliferation. (E) Transwell detection of cell migration. (F) Expression of EMT related proteins was determined by western blot. (G) Tube formation assay tested angiogenesis using HUVECs cultured in conditioned medium from control or KAT2A-overexpressing OSRC-2 cells. Data were shown as mean ± SD from three independent experiments. **P*<0.05, ***P*<0.01, ****P*<0.001. KAT2A: Lysine acetyltransferase 2A. RT-qPCR: Reverse transcription-quantitative polymerase chain reaction. CCK-8: Cell counting kit-8. EMT: Epithelial-mesenchymal transition. HUVECs: Human umbilical vein endothelial cells.
